# Flaxseed extract reduces tissue accumulation and enhances urinary excretion of chondroitin sulphate in the rat: a possible new path in substrate reduction therapy for mucopolysaccharidosis

**DOI:** 10.1080/13880209.2022.2068618

**Published:** 2022-05-30

**Authors:** Sabir Es-said, Karima Lafhal, Abdelaati Elkhiat, Miloud Hammoud, Noureddine Regbaoui, Aicha Ezoubeiri, Rachida Makbal, Safia Sbyea, Omar Elhiba, Souad Sellami, Hanane Rais, Abdallah Karim, Halima Gamrani, Noureddine Rada, Mohammed Bouskraoui, Naima Fdil

**Affiliations:** a Metabolic Platform, Biochemistry Laboratory, Faculty of Medicine, Cadi Ayad, University, Marrakech, Morocco; b Neurosciences, Pharmacology and Environment Unit (NPEU), Faculty of Sciences, Semlalia, Cadi Ayyad University, Marrakech, Morocco; c Department of Biology; Faculty of Science Semlalia, Cadi Ayad University, Marrakesh, Morocco; d Clinical Laboratory; Ibn Tofail Hospital, Mohammed VI Hospital University, Marrakech, Morocco; e Biotechnology, Protection, and Development of Plant Resources Laboratory, Faculty of Sciences, Semlalia, Cadi Ayyad University, Marrakech, Morocco; f Nutritional, Physiopathologies and Toxicology Team, Faculty of Sciences, Chouaib Doukkali University, El Jadida, Morocco; g Department of Pathological Anatomy, Mohammed VI Hospital University, Marrakech, Morocco; h Laboratory of Coordination Chemistry, Faculty of Sciences Semlalia, Cadi Ayad University, Marrakech, Morocco; i Mother-Child Hospital, Pediatric Department, Mohammed VI University Hospital, Cadi Ayad University, Marrakesh, Morocco

**Keywords:** Antioxidant activity, chondroitin 6-sulphate, chelating effect, genistein, quercetin, lignans

## Abstract

**Context:**

Chondroitin 6 sulphate (C6S) is a glycosaminoglycan (GAG) whose accumulation is notable in mucopolysaccharidosis type IVA and VII. Flaxseed, *Linum usitatissimum* L. (Linaceae) (FS), is reported to have comparable properties to those of soybean, a source of genistein, a potential new treatment for MPSs.

**Objective:**

We assess the effect of total ethanol flaxseed extract (EFSE) in an animal model of C6S accumulation.

**Materials and methods:**

The study was performed in adult male Wistar rats (*n* = 24) for 15 successive days. The animals were divided into four groups: (1) control injected with physiological saline buffer, (2) intoxicated rats injected intraperitoneally with C6S, (3) intoxicated with C6S and treated with EFSE, and (4) treated with EFSE. All groups were subjected to histopathological and biochemical studies. The antioxidant and phytochemical properties of EFSE were examined.

**Results:**

Dry EFSE contains total phenols (6.28 mg EAG/g), condensed tannins (2.98 mg ECAT/g) and flavonoids (0.44 mg ECAT/g) with high antioxidant potential [RPE (IC_50_ = 8.37 ± 0.176), DPPH (IC_50_ = 12.79 ± 0.273)]. The LD_50_ is higher than 5000 mg/kg. The histopathological examination showed an accumulation of C6S in the C6S intoxicated group, which disappeared in the C6S-EFSE treated group. GAGs assays showed an increased excretion in the C6S intoxicated group and increased excretion of 14% in the C6S-EFSE group compared to the C6S group.

**Discussion and conclusions:**

EFSE showed significant potential for chelation. Its use for the treatment of GAG accumulation could be suggested and generalized to a larger study population.

## Introduction

Chondroitin 6-sulphate (C6S) is a hydrophobic polysaccharide found naturally in the body. For therapy, it has mostly been used to reduce pain and inflammation, to improve joint function and problems with muscles used for chewing as well as delaying the progression of osteoarthritis. Its hydrocolloid properties confer much of the compressive resistance of cartilage. The deficiency/absence of the enzyme involved in the intra-lysosomal degradation of C6S leads to the accumulation of these glycosaminoglycans (GAGs) in the lysosomes, resulting in cell, tissue, and organ dysfunctions and the urinary excretion of high amounts of C6S. Such abnormalities characterizes a group of diseases under the name of mucopolysaccharidosis (MPS) (Neufeld 2001).

Much light has been shed on the potent therapeutic properties of flaxseed in several diseases including diabetes, heart disease, colorectal, breast, and prostate cancer (Cunnane et al. 1993; Tarpila et al. 2005). Flaxseed is reported to have comparable functional properties to that of soy bean; known for its wealth of oestrogens. Two of these so-called phytoestrogens, lignans and isoflavones, have been suggested to play several biological roles in the chemoprevention of estrogen-dependent breast cancer and colon cancer. This hypothesis has been supported by Peterson and Barnes (1991, 1993). Authors concluded that two isoflavones, genistein and daidzein, inhibit the growth of human breast cancer and prostate cancer cell lines in culture by mechanisms independent of inhibition of the bending of steroids to their receptors.

Genistein has recently been put forward as a potential new treatment for MPS. Several studies (Piotrowska et al. 2006, 2008; Jakóbkiewicz-Banecka et al. 2009) have shown that the treatment of Sanfilippo patients with a genistein-rich soy isoflavone extract (called gene expression-targeted isoflavone therapy) was effective on behavioural and cognitive complications and that an increased dose of genistein may improve the efficacy of the treatment. Friso et al. (2010) reported a decreased GAG storage in the MPSII mouse model following genistein administration. Such results support the use of this isoflavone in a combined therapeutic protocol for the treatment of MPS.

Therapeutic approaches with MPS patients have been performed with pure synthetic genistein at doses varying from 5 to 150 mg/kg/day. In our context, the high cost of genistein therapy, particularly in the developing countries as well as its local availability, limit its use for MPS patients. Isoflavones and lignans have similar therapeutic effects. Flax seeds are a rich source of mammalian lignan precursors, with values 75 to 800 times higher compared to other oilseeds (Jenab and Thompson 1996). Therefore, we opted for the evaluation of the possible effects of the FS ethanol extract on the accumulation of C6S in some vital organs as well as its urinary excretion in rats.

## Material and methods

### Chemicals and reagents

All chemicals used in this study were of analytical grade: C6S was supplied by Sigma Aldrich (Germany) (CAS Number: 39455-18-0), quercetin (≥95%) (CAS Number 117-39-5), gallic acid (≥97%) (CAS Number: 149-91-7), *para*-coumaric acid (≥98%) (CAS Number: 501-98-4), ferulic acid (≥99%) (CAS Number: 537-98-4), sinapic acid (≥98%) (CAS Number: 530-59-6), and tannic acid (≥95%) (CAS Number: 1401-55-4), were purchased from Sigma-Aldrich (Germany), vanillin (99.8%) (Lot: 080417CE) from SOLVACHIM (Casablanca, Maroc). Methanol (≥99%) (CAS Number: 67-56-1), ethanol (≥99%) (CAS Number: 64-59-6) HPLC grade from Merck (Germany).

### Animal protocol

Male Wistar rats were obtained from Central Animal Care at the Faculty of Medicine and Pharmacy, Cadi Ayad University, Marrakesh. All rats were housed in Plexiglas cages under controlled temperature (25 °C), humidity (60%), and lighting (12 h light/dark cycles, dark from 7 P.M.), with *ad libitum* access to food and water. All experiments were performed according to the guidelines for the use of laboratory animals described in the Scientific Procedures on Living Animals ACT 1986 (European Council directive: 86/609/EEC) and approved by Moroccan Society for Animals Research and supervised by its Committee for the Ethical care of Animals based at the Marrakesh Faculty of Medicine. Thus, all efforts were made to minimize the number and reduce the suffering of animals.

**Figure 1.**
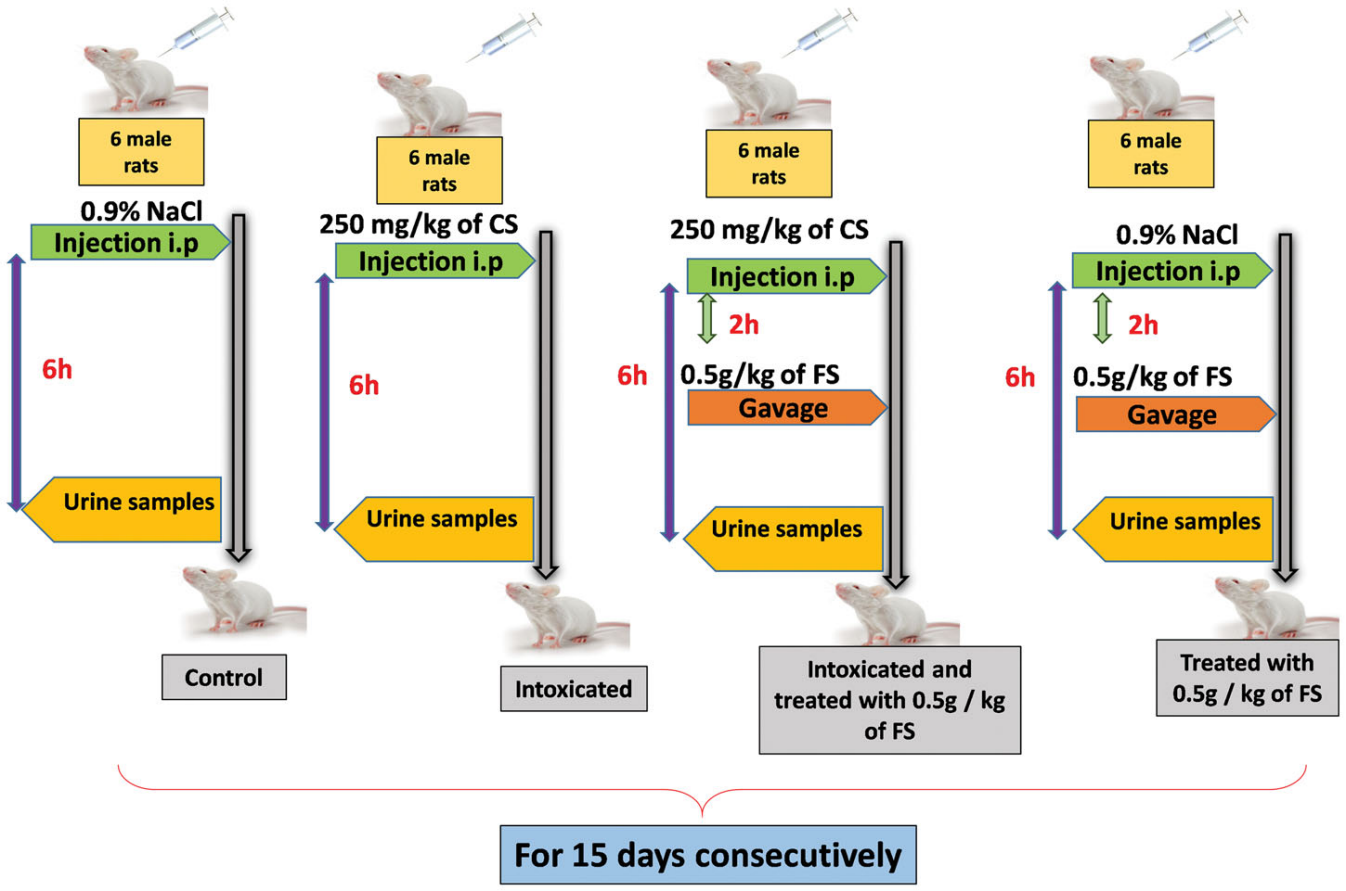
Study design adopted with groups of rats included in our study. i.p: intraperitonial; CS: chondroitin 6-sulphate; FS: flaxseed.

Male rats weighing 200-250 g were divided into 4 groups. **Group I**: control rats (CTR) (*n* = 6) injected i.p with physiological saline buffer (0.9% NaCl) during 15 days consecutively. **Group II**: C6S treated rats (224.2 ± 3.9 g SEM, *n* = 6) injected i.p. with C6S at a dose of 250 mg/kg B.W, for 15 consecutive days. **Group III**: C6S + FS (249.6 ± 7.3, *n* = 6), received, in addition to C6S (250 mg/kg B.W, i.p), an aqueous solution of total (flaxseed) FS extract at a dose of 500 mg/kg B.W per os 2 h following C6S administration during 15 days. **Group IV**: FS (240 ± 7.7, *n* = 6), received only total FS extract solution at a dose of 500 mg/kg B.W per os, and injected i.p. with physiological saline buffer (NaCl 0.9%) for 15 days. The experimental protocol and the doses of C6S and FS used are based on previous study and literature reports (De los Reyes et al. 2000; Bauerova et al. 2011; Shimada et al. 2014; Latha et al. 2016; Miraglia et al. 2016). Gavages and injections were performed between 10 and 12 a.m. For oral administration, the total FS extract, taking into account its water solubility, was dissolved in distilled water. For the purposes of intraperitonial injections, CS was dissolved also in distilled water. Study design is shown schematically in Figure 1.

### Collection of plant material and preparation of total extracts

*Linum usitatissimum* L (Linaceae), locally named ‘zariit kettane’, was collected in April 2020 from the Provence of Essaouira, Lakrimate village, geographic coordinates (31°32′20.8″N; 9°24′24.1″W), Morocco. The plant material was botanically classified and its correct botanical identification was authenticated by Professor Ouhammou Ahmed (Laboratory of Environment and Ecology (L 2 E, CNRST Associated Research Unit, URAC 32), Regional Herbarium MARK, Faculty of Sciences Semlalia, Cadi Ayad University, Marrakesh, Morocco). A voucher specimen of the plant was deposited in the herbarium of the Semlalia Science Faculty, Cadi Ayad University (voucher number: 13293).

Seeds were crushed at ambient temperature and stored at 4 °C prior to use. Freshly ground flaxseeds (200 g) and 800 mL of pure ethanol solvent 98% were introduced into a 1.5 L Erlenmeyer flask. The mixture was then macerated and left at room temperature in the dark for 48 h. Afterwards, filtration was carried out, and the filtrates were subjected to evaporation under vacuum using a Rotavapor (BUCHI Rotavapor R200) at 45 °C. The extract obtained was refrigerated (4 °C) until further use. Maceration helped us to extract the flaxseed compounds groups according to their solubility in the different organic solvents. The solvent used in this study was the ethanol, which is known for its ability to extract the maximum number of secondary metabolites present in the seed. Extraction was done following the method of Anwar and Przybylski (2012) with slight modifications.

### Acute toxicity study

In order to assess the lethal effect of FS extract, acute toxicity of the FS was assessed according to the Organization for Economic Co-operation and Development (Oecd/Ocde) guidelines 425 (2008). Indeed, Wistar rats (6 rats per group, sex-ratio 1:1) weighing (200–250 g) received the total FS extract p.o at different doses (0.5, 1, 2, 3.5, 5 g/kg) for 15 days. Animals were observed daily for symptoms of toxicity, body weight changes and mortality.

### Identification of secondary metabolites

Common phytochemical tests were carried out on the ethanol extract to ascertain the presence of some major natural chemical groups according to the protocol described by Harborne (1980). The qualitative presence of the following secondary metabolites was analysed: flavonoids, steroids, terpenes, tannins, coumarins, saponins, and alkaloids.

### Determination of total phenolic compounds

The total phenolic content of FS ethanol extract was assessed using Folin-Ciocalteu method Singleton and Rossi (1965). Briefly, 100 µL of the extract was diluted with 3.7 mL of distilled water, and 200 µL of Folin-Ciocalteu reagent was added. Three minutes later, 20% sodium carbonate (1 mL, Na_2_CO_3_) was added. The solution was agitated and incubated in dark for 45 min at 25 °C. Then, absorbance was measured at 725 nm using a UV-V spectrophotometer (VR-2000, Spain). The total phenolic concentration of FS ethanol extract was reported in terms of mg equivalent Gallic acid per g of the dry matter of extract (DM). All samples were carried out in triplicate.

### Determination of total flavonoids

Flavonoids content was determined by aluminium trichloride (AlCl_3_) method using catechin as reference compound (Zhishen et al. 1999). Briefly, 200 µL aliquots of diluted extract were added to 5% NaNO_2_ solution (60 µL), followed by 10% AlCl_3_ (40 µL), and incubated for 6 min, followed by the addition of NaOH (400 µL). Then, distilled water (500 µL) was added. The absorbance of the solution was measured at 510 nm. Results were expressed as catechin equivalents per gram of extract (CAT/g DM).

### Determination of total tannins

Tannins in FS extract were evaluated as follows (Xu and Chang 2007): sample extract (300 µL) was added to a solution of vanillin (3 mL, 4% methanol) and HCl (1 mL). The absorbance was measured at 500 nm. Results were reported as catechin per g of the extract.

## *In vitro* antioxidant activity

### Determination of the free radical scavenging activity by the 2,2-diphenyl-1-picrylhydrazyl (DPPH) free-radical scavenging assay

This method based on measuring the capacity of the extract to trap the DPPH radical (Burda and Oleszek 2001). The different sample or standard concentrations (25 µL) (butylated hydroxyl toluene (BHT) and quercetin) were added to 2.8 mL of the methanol solution of DPPH (0.004%) and left to stand 60 min at room temperature. The absorbance was recorded at 517 nm. Assays were carried out in triplicate. The percentage inhibition (1%) of the DPPH radical was calculated by the following equation:

$$\text{Inhibition}\;\left(\%\right)=[\left({\text{control}}_{\text{absorbance}}-{\text{sample}}_{\text{absorbance}}\right)/{\text{control}}_{\text{absorbance}}]\times 100$$

### Reducing power assay

The reducing power was based on Fe (III) to Fe (II) transformation in the presence of the solvent fractions. This assay was realized using the method of Oyaizu (1986). The plant (1 mL) was added to the sodium phosphate buffer solution (0.2 M, pH 6.6, 2.5 mL) and 1% of potassium ferricyanide K_3_Fe (CN)_6_ (2.5 mL). After 20 min of incubation at 50 °C, Cl_3_CCOOH (2 mL) was added and centrifuged (3000 *g*, 10 min). Finally, a 2.5 mL aliquot of the mixture was combined with 0.5 mL ferric chloride (0.1%, FeCl_3_) and 2.5 mL of water. Reading absorbance was set at 700 nm.

BHT and quercetin were used as positive controls. The increase of absorbance in the reaction medium indicates the increase of the iron reduction. Concentration IC_50_, defined as the concentration of antioxidants required to reduce 50% of the initial concentration of ferric thiocyanate, is an index used to compare and express the reducing power of bioactive substances.

### ABTS radical scavenging assay

Free radical scavenging activity of plant samples was determined by ABTS radical cation decolonization assay (Re et al. 1999). ABTS^+^ cation radical was produced by the reaction between 7 mM ABTS in water and 2.45 mM potassium persulfate (1:1) and stored in the dark at room temperature for 12–16 h before use. ABTS^+^ solution was then diluted with methanol to obtain an absorbance of 0.700 at 734 nm. After the addition of 5 μL of plant extract to 3.995 mL of diluted ABTS^+^ solution, the absorbance was measured at 30 min after the initial mixing. An appropriate solvent blank was run in each assay. All the measurements were carried out at least three times. Inhibition of absorbance at 734 nm was calculated using the formula: ABTS^+^ scavenging effect (%) = ((AB–AA)/AB) ×100 (2), where AB is absorbance of ABTS radical + methanol, AA is absorbance of ABTS radical + sample extract/standard. Trolox was used as standard substance.

### Determination of polyphenols compounds in FS extracts by UHPLC analysis

The ethanol extract of FS was analysed for its content of marker compound, flavonoids, and polyphenols by HPLC method. HPLC analysis was performed on KNAUER apparatus provided by Centre for Analysis and Characterization (Cadi Ayyad University, Faculty of Science Semlalia, Morocco) with a column (Eurospher II 100-5 C18, 250 × 4.6 mm) protected by Agilent technologies RP-18 (10 mm × 4.6 mm) pre-column. Both columns were placed in a column oven set at 25 °C. Two solvents were used with a constant flow rate of 1 mL/min in gradient program (Table 1) (Eluent A = Formic acid 0.1%, Eluent B = Methanol). The injection volume was 10 μL. HPLC analysis was first performed with the standards then followed by the FS extracts and finally spiking the samples with the standards. The phenolic compounds are characterized according to their UV–Vis diode array detector at 280 nm spectrum, and they were identified by comparing with standard of each compound using the retention time (RT). The UV/Vis detector (wavelength scanning range 200–700 nm). All the solvents used were of HPLC grade.

**Table 1.** Gradient time table.

Time [min]	A [%]	B [%]	Flow [mL/min]
0	95	5	1
3	75	25	1
6	75	25	1
9	63	37	1
13	63	37	1
18	46	54	1
22	46	54	1
26	5	95	1
29	5	95	1
30	95	5	1

## Biochemical analysis

### Plasma markers of liver and kidney function

Blood samples were collected from the jugular vein (under anesthesia) from each animal from each group in pre-labeled centrifuge tubes, and allowed to clot for 20 min at room temperature. Serum was separated by centrifugation at 3000 rpm for 20 min. Total bilirubin, aspartate aminotransferase (ASAT), alanine transaminase (ALAT), alkaline phosphatase (AP) and γ-glutamyl transpeptidase (GT), urea, and creatinine levels were measured in sera of all groups using CHRONOLAB kits applied to BA-88A Semi-Auto Chemistry Analyser (Mindray-China).

### Urinary GAG content

Urine was collected every day for all groups, 6 h post injection of FS extract in the metabolic cages (Nalgene, Thermo Fisher) consist of a circular upper portion, which houses the rat; a wire-grid floor (diameter, 21.5 cm; approximate surface area, 363 cm^2^; opening, 1 × 3.1 cm); and a lower collection chamber with a specialized funnel that separates faecal pellets and urine that fall through the grid floor for their collection into 2 separate tubes (diameter, 4 cm; Nalgene, Thermo Fisher) (Hoffman et al. 2018). Urinary GAG content (calculated as mg GAG per mmol creatinine) was determined using the protocol described by De Jong et al. (1989) with slight modifications (Fdil et al. 2020; Sabir et al. 2020); GAG content was determined in urine samples mixed with 1, 9-dimethylmethylene blue chloride solution (Sigma-Aldrich) in a quartz cell. The mixture is shaken and allowed to stand for 5 min. The measurements were performed using a Jasco750 Diode Array Spectrophotometer (Shimadzu Technologies, Japan). Afterwards, the absorption spectrum is recorded between 400 and 800 nm. The difference between the maximum and the minimum absorption (525 and 595 nm, respectively) is recorded. A standard curve was prepared using chondroitin sulphate type (Sigma-Aldrich). Urinary creatinine was measured by a Cobas c 311 analyser – Roche Diagnostics.

### Liver, kidney and spleen morphometric analysis

For each rat of each group, liver, kidney and spleen were entirely and carefully dissected and freshly weighted in analytical balance (KERN & SOHN GmbH. D-72336 Balingen in Germany). The organ-body weight ratio (OBWR) was calculated as:

OBWR=(organ weight/body weight at the moment of sacrifice)×100. Later, livers and kidneys were post−fixed in formalin solution (4%) for the histological study.

### Histological study

The histopathological analysis was performed in the Anatomy and Pathological Cytology department, CHU Mohamed VI, Marrakesh. Following 24 h post-fixation, different samples from the liver and kidney tissues were taken and placed in plastic cassettes and then placed in circulating automaton during 18 h (Leica ASP 300 S). Wherein, tissues are dehydrated in ethanol baths with increased concentrations (70%, 95% and 100%), followed by immersion in toluene baths. Later, the tissue samples were imbibed with paraffin. The final step of coating consists of including the impregnated tissue in a paraffin block, which by solidifying will allow its cutting. The paraffin block containing the tissue is cut into fine ribbons (4 μm thickness) using a semi-automatic microtome (Leica Biosystems RM 2245). The specimens were stained with a standard Hematoxylin-Eosin (HE) stain and Alcian Blue (Friso et al. 2010), staining (staining of GAG deposits), then mounted on slides for a histological examination by two blind anatomo-pathologists. Photos of liver and kidney tissues were taken by an appropriate microscope (Carl Zeiss, Oberkochen, Germany) coupled to High resolution Camera (Nikon, Coolpix P7100 Made in Indonesia) (El Khiat et al. 2019).

### Statistical analysis

All the results were expressed as the mean ± standard errors of mean (SEM). Comparison between different groups was made using the one-way analysis (ANOVA) and repeated measure model of ANOVA followed by a *post hoc* Tukey test. A value of *p* < 0.05 was considered to be statistically significant. The SigmaPlot 12.5 software was used for statistical analysis.

## Results

### Yield test and acute oral toxicity of plant extracts

The yield of ethanol extract was 7.9% for FS. The rats tolerated all doses (0.5, 1, 2, 3.5, 5 g/kg) of FS ethanol extract, no mortality was recorded, and no external symptoms of toxicity were observed within the period of treatment with an LD_50_ value greater than 5 g/kg.

### Phytochemical screening of ethanol extract of flaxseed

Phytochemical analysis revealed the presence of flavonoids, anthocyanin, tannins, quinones, leuco-anthocyanin, saponins, terpenes and steroids (Table 2).

**Table 2.** Phytochemical screening on the ethanolic extract of *Linum usitatissimum*.

Metabolites	Flaxseed extract
Flavonoids	++
Anthocyanin	+
tannins	+
Quinones	+
Terpenes and steroids	+++
Leuco-anthocyanim	+
Saponins	++

Test results are classified as strongly positive (+++), moderately positive (++), weakly positive (+).

### Total phenol, flavonoid and condensed tannins levels

**Figure 2.**
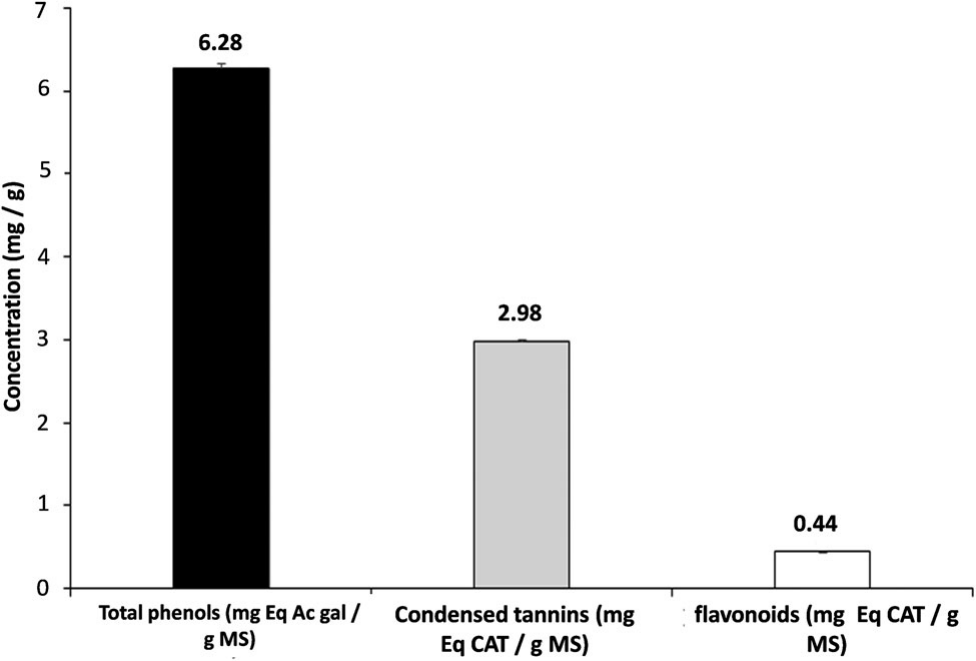
Total phenolic compounds, total tannins, and total flavonoids compounds of flaxseed ethanolic extract.

The quantitative analysis in total phenols, condensed tannins, and flavonoids shows that the ethanol extract contains total phenols, condensed tannins, and flavonoids levels of 6.28 mg EAG/g dry extract, 2.98 mg ECAT/g and 0.44 mg ECAT/g dry extract, respectively (Figure 2).

### Antioxidant activity DPPH, reducing power and ABTS

The measurement of the antioxidant activities of the extract was performed in relation to the latter’s IC_50_ concentration which expresses the concentration’s capacity of the samples to reduce the initial DPPH absorbance by 50%. The concentrations that led to IC_50_ are given in Table 3. The lower IC_50_ values reflected better protective action. The results showed that FS ethanol extract exhibited interesting antioxidant activity. The lowest IC_50_ was obtained with reducing power assay (IC_50_ = 8.37 ± 0.176), followed by DPPH (IC_50_ = 12.79 ± 0.273). Whereas FS ethanol extract displayed a great antioxidant activity in ABTS test system with IC_50_ values of (495.95 ± 8.02). Compared with quercetin and butylated hydroxytoluene (BHT) the IC_50_ values of FS ethanol extract were less effective than those of synthetic antioxidant agent.

**Table 3.** Antioxidant activity of FS ethanolic extract against DPPH, ABTS and reducing power (PR) methods.

	Extract FSaq (IC_50_ (mg/mL))	quercetin (IC_50_ (mg/mL))	BHT (IC_50_ (mg/mL))	Vitamin C (IC_50_ (mg/mL))
DPPH	12.79 ± 0.273	0.1 ± 0.002	0.3 ± 0.02	0.15 ± 0.003
PR	8.37 ± 0.176	0.06 ± 0.001	0.05 ± 0.001	0.013 ± 0.0009
ABTS	495.95 ± 8.02	0.05 ± 0.01	0.65 ± 0.004	2.635 ± 0.009

Values are expressed as mg per ml and represent means of triplicate determination (mean ± SEM).

Values represent means ± standard errors of mean (SEM) for triplicate experiments.

### HPLC analysis of the ethanol extract of FS

**Figure 3.**
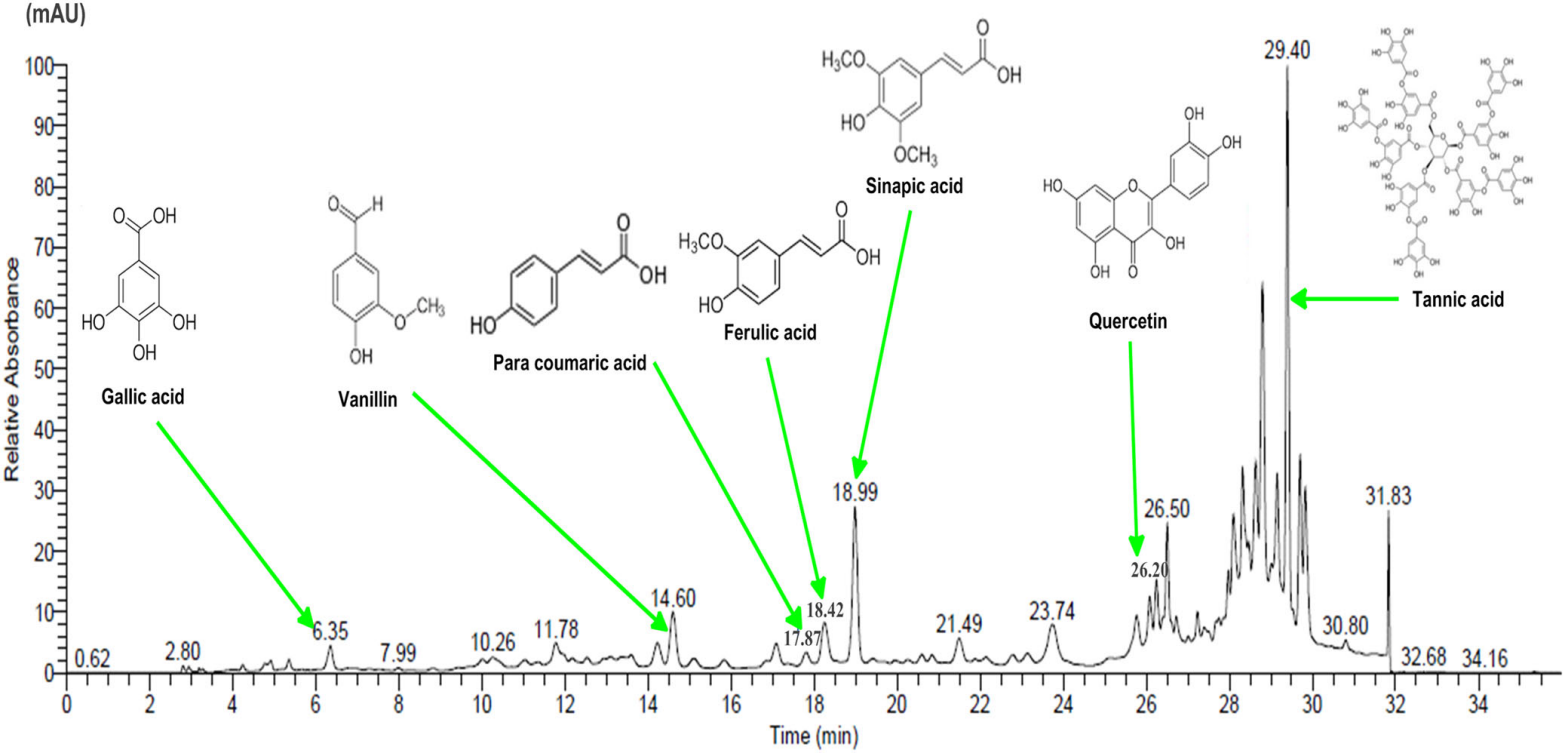
HPLC chromatogram recorded at 280 nm for the main phenolic compounds identified in the FS.

Identification of phenolic compounds in FS extract was performed by using the UHPLC, analysis revealed the presence of poly-phenolic compounds. On the basis of retention time (min) of standard compounds, the polyphenols identified in FS were gallic acid (Rt = 6.35), vanillin (Rt = 14.60), para coumaric acid (Rt = 17.87), ferulic acid (Rt = 18.42), sinapic acid (Rt = 18.99), quercetin (Rt = 26.20), and tannic acid (Rt = 29.40) (Figure 3). However, some of the polyphenols could not be identified due to lack of standards. Tannic acid and sinapic acid were the most represented compounds in the FS ethanol extract.

### Morphometric analysis

**Figure 4.**
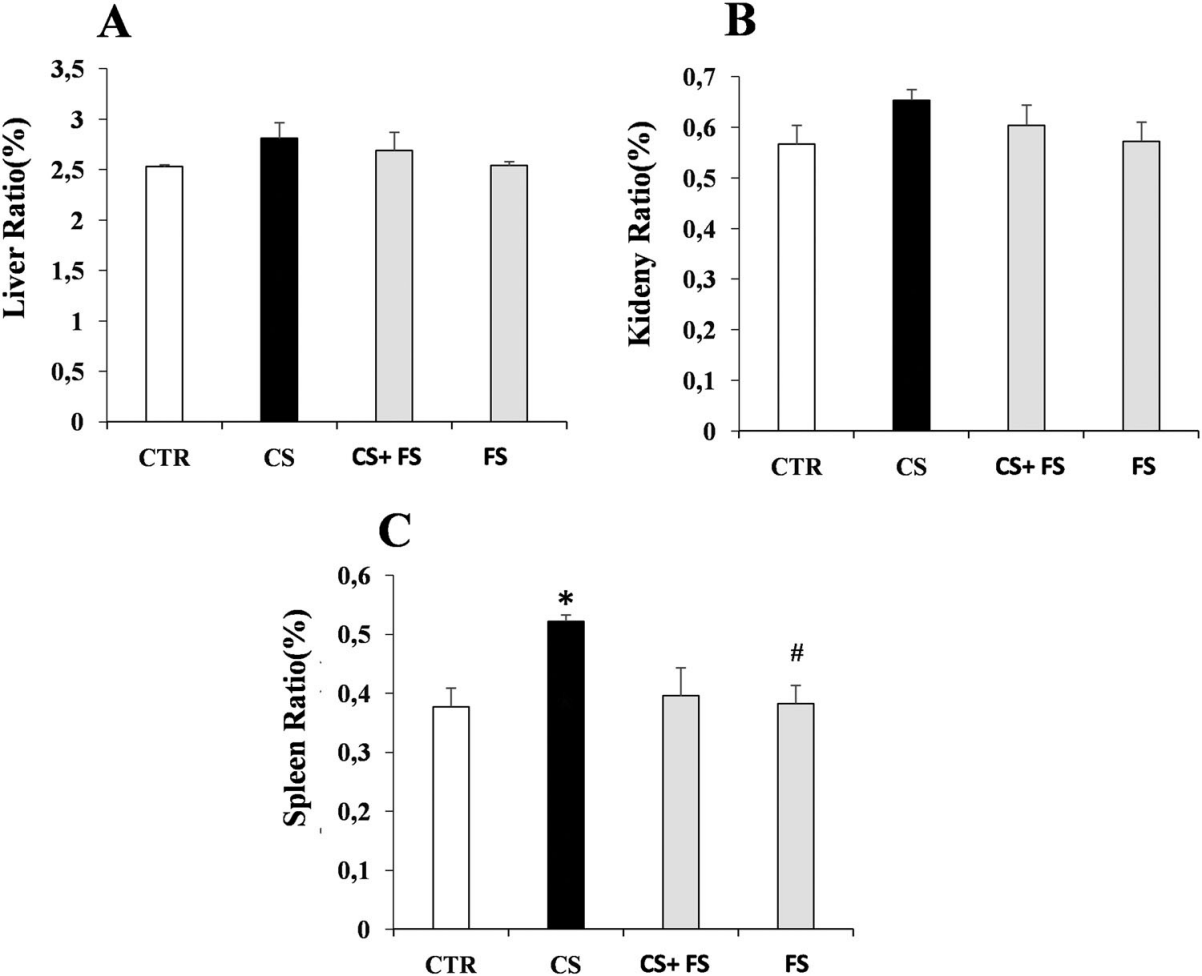
Histograms showing the OBWR of the liver, the kidney and the spleen of the intoxicated rats (CS) and rat treated with 500 mg/kg extract of Flaxseed vs. control rat (CTR) and rat treated only with 0.5 mg /kg extract of flaxseed. 6 rats per group were used and a *p*-value *p* < 0.05 is considered as a statistically significant difference. *p < 0.05 vs CTR, #*p* < 0.05 vs CS.

Comparison of the OBWR for the liver, the kidney and the spleen between the different groups, showed a slight and non-significant increment of the liver (Figure 4(A)) and kidney ratios (Figure 4(B)) in the C6S group compared to the control, C6S + FS and FS groups. While the spleen, contrarily to the other organs, exhibits a significant increase in its ratio (Figure 4(C)) in the C6S group compared to the: control (*q* = 44.88; *p* = 0.022), and C6S + FS (*q* = 45.52; *p* = 0.010).

## Biochemical analysis

### Plasma markers of liver and kidney function

Besides the morphometric analysis of the liver, kidney, and spleen, we performed biochemical assays for plasma markers in rats from all groups. Based on the results obtained, we found a non-significant increase (*p* > 0.05) in ALAT, ASAT, alkaline phosphatase, urea, and creatinine in the C6S intoxicated rats compared to the control group, this slight increase was corrected in the group treated with 500 mg of the extract (Table 4). Data are reported as mean ± SEM. Data was subjected to a one-way analysis of variance (ANOVA). A value of *p* < 0.05 was considered to indicate statistical significance between control and treated groups.

**Table 4.** The enzymatic activities in sera of intoxicated rats (CS), rats treated with 0.5 g/Kg (CS + 0.5) of flaxseed extract after CS intoxication and rats treated only with 0.5 g /kg extract of flaxseed and control rats (CTR).

Parameters	CTR	CS	CS + FS	FS
ALT (U/L)	45.33 ± 1.25	53.33 ± 1.25	44.66 ± 1.44	49.00 ± 0.86
AST (U/L)	152.33 ± 1.60	158.00 ± 3.60	154.50 ± 3.79	155.00 ± 1.41
Alkaline phosphatase (U/L)	125.25 ± 2.53	128.50 ± 2.25	126.00 ± 2.74	126.67 ± 2.40
Urea (g/L)	0.32 ± 0.03	0.36 ± 0.06	0.32 ± 0.03	0.32 ± 0.04
Creatinine (U/L)	3.00 ± 00	3.50 ± 0.29	3.25 ± 0.25	3.00 ± 0.00

Our data demonstrated that 250 mg/kg of C6S injection induced a slight increase in enzymatic activities of ALAT, ASAT, and ALP activities.

### Urinary GAG (C6S) assays

**Figure 5.**
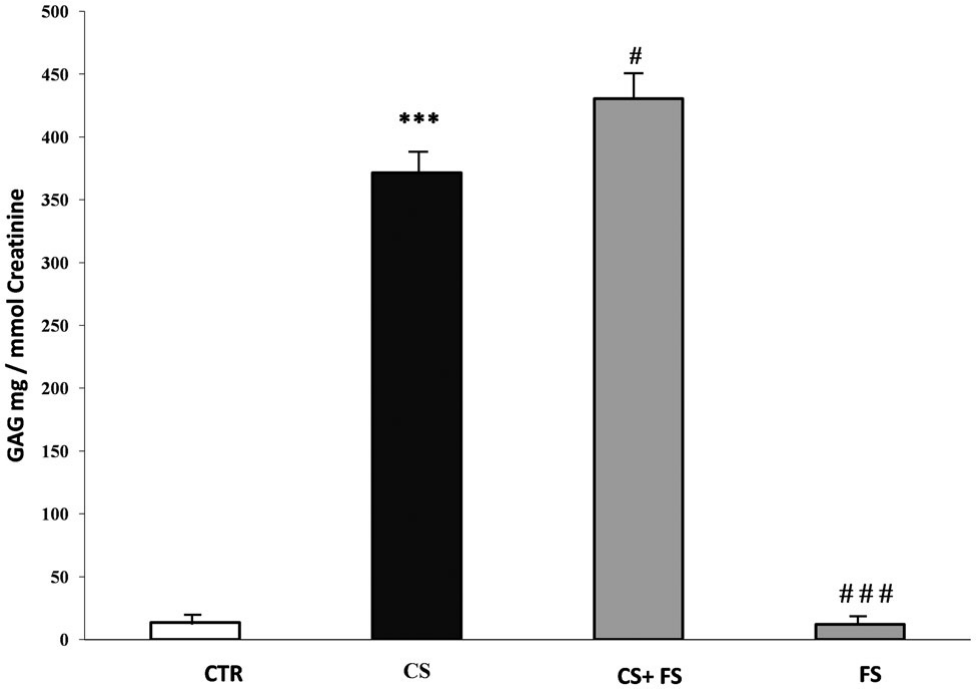
Urinary glycosaminoglycans (GAG) levels in mg/mmol of creatinine in rats of the different groups studied. Control, addicted to CS, intoxicated and treated with ethanolic extract of FS 500 mg (CS + 500 mg), and treated with extract 500 mg alone. Urinary GAG levels were significantly decreased in rats treated with 500 mg kg-1·day-1 for 15 days. 6 urine samples were used from each group and a p-value *p* < 0.05 is considered a statistically significant difference. ***p < 0.001 vs CTR, ###*p* < 0.001vs CS.

The administration of the chondroitin 6-sulphate resulted in an increased amount of C6S in urine. Based on the GAG assay results using DMB method, we noted a significant increase of GAGs excreted in the C6S group compared to the control (*q* = 3.27; *p* < 0.05); treated intoxicated rats compared to the control (*q* = 5.69; *p* < 0.05) and the treated intoxicated rats compared to C6S intoxicated rats (*q* = 2.41; *p* < 0.05). Figure 5 shows urinary GAG content in each animal’s group. For six out of six treated C6S intoxicated rats, there was an increase of urinary GAG up to 14% compared to urinary extracted GAG in C6S intoxicated rat group.

## Histopathological analysis

### Staining with hematoxylin-eosin (H & E)

**Figure 6.**
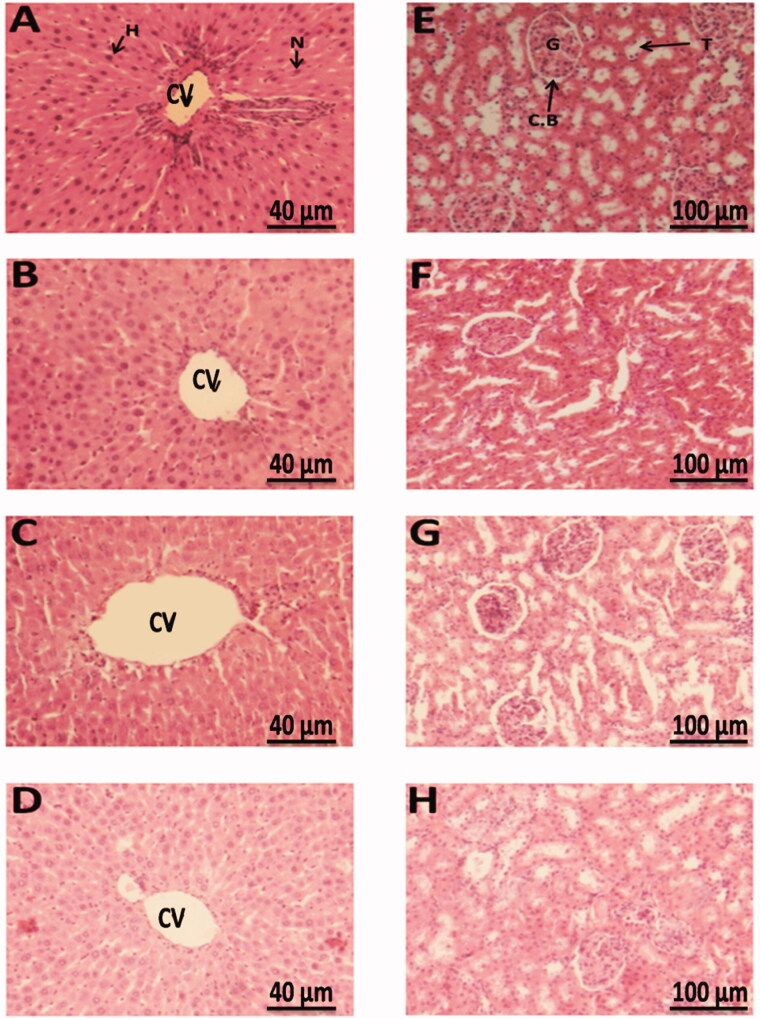
Photomicrographs of the liver and kidney tissues stained with H&E performed after 15 days’ treatment. Representative sections of liver (A–D), kidney (E–H), include: (A and E): control, (C and G): overloaded by CS and treated with 0.5 g•kg-1•day-1 of the FS total ethanolic extract, (B and F): intoxicated with CS, (D and H): treated rats alone with 0.5 g/kg/dayof the FS total ethanolic extract. 6 samples of liver and kidneys were studied from each group. Central vein (CV), Core (N), Hepatocyte (H). Glomerulus (G), Bowman capsule (C.B), Tubule (T). Scale bar, 100 µm.

In order to assess the eventual histological damages of the liver and the kidney tissues resulting from CS intoxication, a histopathological analysis through H & E staining was performed. Indeed, microscopic examination of the liver tissue showed in all the studied groups preserved normal hepatic lobular architecture with normal hepatocytes arranged in thin plates. The central vein was normal and surrounded by distinct hepatocytes and normal sinusoidal episodes (Figure 6(A–D)) and for all groups, we noted the absence of inflammatory and hepatocytes degenerative features. The kidney tissue as well, showed normal architecture of renal tissue, glomerulus, and renal tubules with absence of cell necrosis (Figure 6(E–H)).

### Alcian blue staining to track the GAG deposits in visceral organs

**Figure 7.**
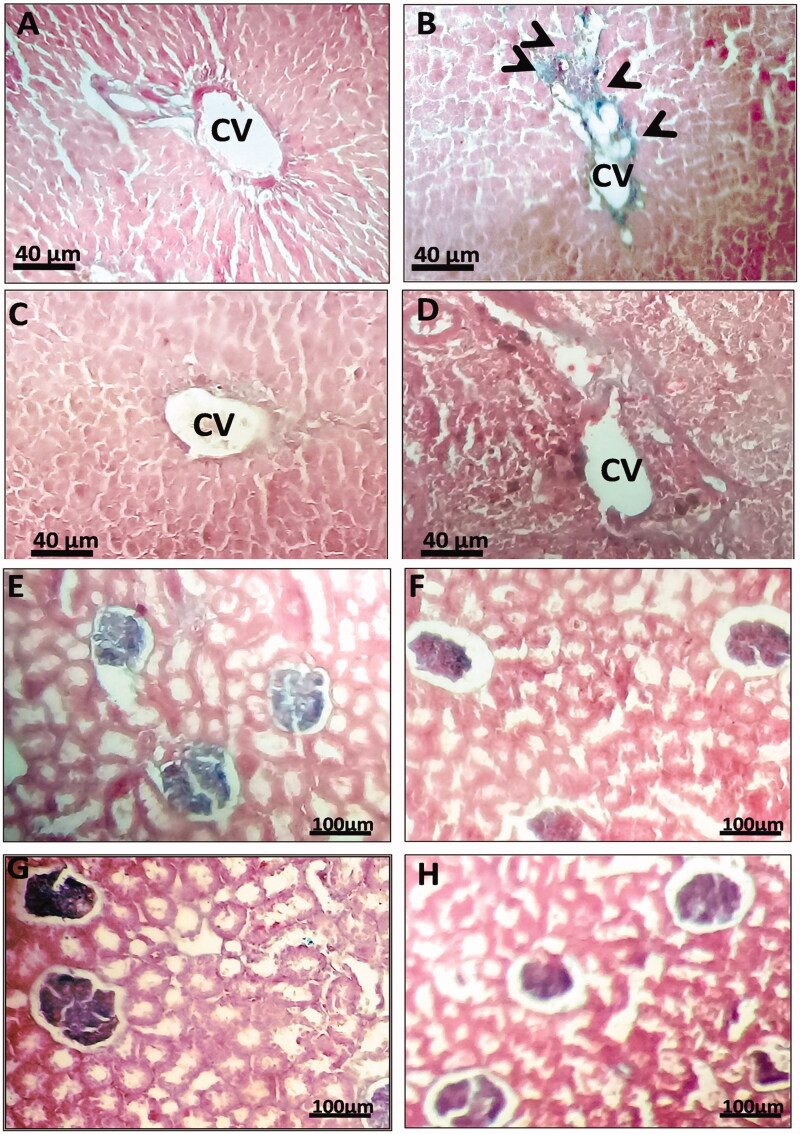
Photomicrographs of the liver and kidney tissues stained with Alcian blue for glycosaminoglycans (GAG) performed after 15 days treatment. Representative sections of liver (A–D), kidney (E–H), include samples from untreated rats (B and F), intoxicated FS-treated rats 0.5 g/kg/day (C and G), FS-treated rats alone 500 g/kg/day (D and H). Arrows indicate GAG deposits. Sections (7 µm) were stained with 1% Alcian Blue, counterstained with 0.1% Nuclear Fast Red. 6 samples of liver and kidneys were studied from each group. Scale bar, 100 µm.

The special staining for GAG deposits using Alcian Blue staining (ABS) (Figure 7) showed in the C6S intoxicated rats, compared to controls, an accumulation of undegraded C6S (blue color), particularly surrounding the central vein of the hepatic lobules (Figure 7(B)), Table 5). Some hepatic lobules showed vacuolated structure. However, treatment with FS (500 mg/kg/day) (Figure 7(C)), seems to restore remarkably such tissular abnormalities. Accumulated GAG in the liver tissue disappeared completely, while the central vein appears normal without any surrounding blue staining (Figure 7(A–D)). Otherwise, FS group appears to be similar to the control group. The preserved histological patterns sustain the absence of any possible hepatotoxic effect of FS ethanol extract (Figure 7(A–D), Table 5).

**Table 5.** Visual qualitative estimation of chondruitine-6-sulphate (CS) deposition in the liver and the kidney tissues stained with Alcian Blue.

	C6S deposition (presence/absence)	
Groups	Kidney	Liver
CTR	–	–
CS	–	+
CS + FS	–	–
FS	–	–

(–) Absence; (+) Presence.

In the kidneys, ABS shows the absence of GAG deposits within the glomerular structures and parenchyma in the CS group compared to controls and the C6S + FS group (Figure 7(E–H), Table 5).

## Discussion

Treatment with FS extract appears to be safe and well-tolerated by animals, and no secondary effects were observed concerning the liver and kidneys, as hepatic and renal assessment were similar to those of control rats. Previous studies also reported that FS had good tolerability and a good safety profile (Ward et al. 2001).

**Figure 8.**
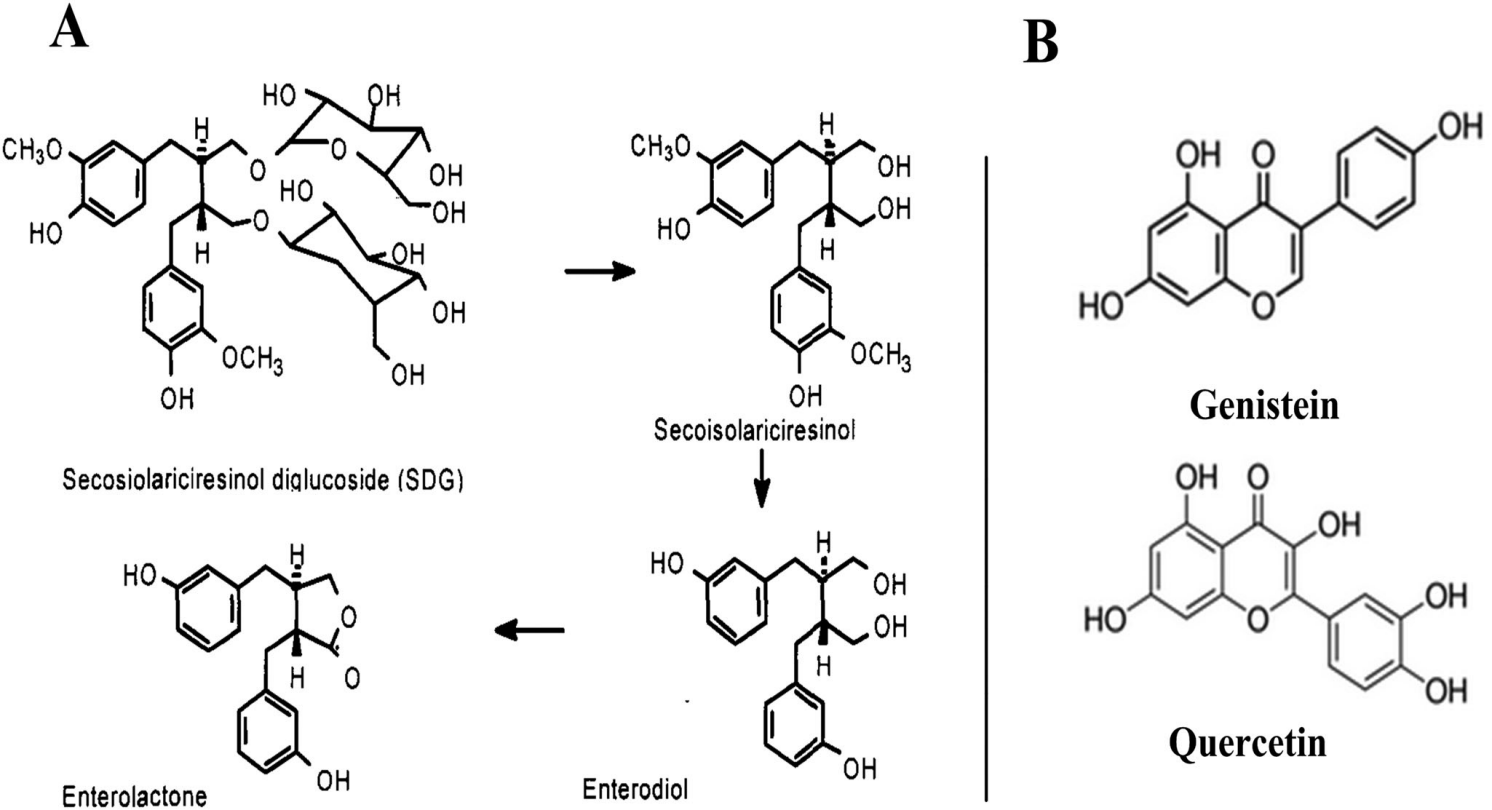
A: Metabolism of SDG by human intestinal bacteria adapted from Muir et al. (1997), B: Similarity of the molecular structures of genistein and quercetin.

Flaxseed is considered as one of the plants that produces mammalian lignans precursors (9–30 mg/g of defatted meal) (Muir et al. 1997). It is a particularly rich source of lignans called secoisolariciresinol glycoside (SDG) and secoisolariciresinol (SECO), the aglycone (non-sugar) portion of SDG. SDG is a plant lignan that is converted by bacteria in the colon of humans (and other animal also) to mammalian lignans known as enterodiol and enterolactone (Figure 8) (Muir et al. 1997). Taking into account these data as well as the encouraging results obtained in MPS III patients treated with genistein; we have undertaken the evaluation of the pharmacological effects of total FS extract in the case of subacute chondroitin sulphate overload in rats. We started by evaluating both the overall chemical composition of the FS and its phenolic and secondary metabolites content, and the antioxidant activity of the ethanol extract. Phytochemical analysis performed on our extract revealed that the plant is rich in secondary metabolites such as flavonoids, tannins, anthocyanins, saponins, quinones, leuco-anthocyanin, steroids, and terpenes. These results are similar to those obtained by the phytochemical study carried out by Beroual et al. (2017).

To evaluate the antioxidant activity of our extract, three different methods were used (DPPH test, Reducing power and ABTS). Data are similar to those of Bekal and Kumari (2015). The presence in flaxseed of tocopherols (vitamin E), β-carotene, phenolic compounds (such as lignans, flavonoids, phenolic acids, tannins and phenylpropanoids) are responsible for its high antioxidant activity.

To the best of our knowledge, this is the first experimental investigation related to the total extract of FS efficiency in the case of GAG (C6S) storage. In this study, the biochemical assays relating to plasma markers in rats showed a non-significant increase (*p* > 0.05) in ALAT, ASAT, alkaline phosphatase, urea and creatinine in groups intoxicated with C6S compared to the control group and the C6S treated group. This is in perfect correlation with the profile of patients with MPS where the disturbance of the hepatic and renal function does not take place. In C6S intoxicated rats, the weight of the spleen has increased, which is correlated with the enlarged spleen described in patients with MPS. This; indicates that the C6S intoxicated rats simulated the majority of the symptoms detected in patients suffering from MPS.

We present data showing that treatment by total extract of FS can significantly reduce the GAG (C6S) content and the size of lysosomal compartment within liver of rats overloaded by C6S at a dose of 250 mg/kg/day (Figure 7(A–D)). Therefore, the plant extract increased significantly the excretion of chondroitin 6-sulphate administered 6 h before the harvest of urine. FS extract seems to chelate C6S excess in treated rats. In FS treated intoxicated rats, the size of visceral organs is not statistically different from those of the control group, demonstrating the effectiveness of the FS extract in neutralizing the C6S accumulated in the organs. Several mechanisms are proposed that can be further investigated by in-depth studies:

- Our extract is rich in lignans (SDG) and its derivatives (Figure 8(A)). These biologically active molecules may have a chelating effect that increases observed C6S excretion, which significantly reduced GAGs accumulation in the tissues. This makes our extract a high effective substrate reducer. Nevertheless, because flaxseed is also rich in dietary fibre and α-linolenic acid, which also have health benefits, the effect of flaxseed cannot be attributed solely to the lignans.
- HPLC analysis revealed the presence of quercetin, *p*-coumaric acid, and several other metabolites, which could explain the neutralizing power of this plant. Indeed, quercetin is a well-known antioxidant that induces protection against various diseases such as osteoporosis, certain forms of cancer, pulmonary, and cardiovascular diseases (Boots et al. 2008). Quercetin, whose structure is similar to that of genistein (Figure 8(B)), may have the same substrate reduction properties as genistein and could be useful in the case of C6S overload. This active metabolite could be a potential candidate contributing to the C6S chelating effect of the FS ethanol extract.
- The richness of flaxseed in tocopherols may have beneficial effects on rats overloaded with C6S taking into account a recent disclosure describing a method for treating lysosomal storage disorders comprising the administration of δ-tocopherol (Zhishen et al. 1999).

Therefore, the total extract of FS could help in solving the present limitations of the available treatment. Indeed, at present, the available therapeutic options for MPS are mainly based on the administration of the extremely expensive recombinant enzymes (ERT). However, while this therapy is effective in reduction of many somatic symptoms, its efficacy in the treatment of the central nervous system is negligible because of problems with crossing the blood-brain-barrier. On another side, the FS extract could provide hope for those forms of MPS for which ERT is not yet available. Because of these potential benefits, the total extract of FS merits a full evaluation in terms of efficacy.

## Conclusions

The present study explores, for the first time, the ability of total FS ethanol extract to prevent the accumulation of C6S in rats through a chelating effect. This treatment prevents lysosomal storage of GAGs (C6S) and may offer a novel therapeutic approach for the management of storage disorders like MPS disease. This effect can be attributed both to the antioxidant activity of flaxseed and to its composition rich in lignans, tocopherols, and quercetin. Other investigations are underway, in our laboratory, for the exact identification of the active compound(s) which would be good candidates for an alternative and/or a combined therapeutic protocol in the management of patients with MPS.
